# In Vivo Acute and Subchronic Toxicity of *Stereospermum acuminatissimum* Stem Bark Aqueous Extract

**DOI:** 10.1155/jt/8985688

**Published:** 2026-10-06

**Authors:** Fabrice Mba Medou, Lucienne Armelle Brooks, Nyemb Nyunaï, Leonel Ntepe Mbah, Esther Ngo Lemba Tom, Theodora Kowa Kopa, Rosette Megnekou

**Affiliations:** ^1^ Centre for Research on Health and Priority Pathologies Institute of Medical Research and Medicinal Plants Studies Yaoundé Cameroon impm-cameroun.org; ^2^ Higher Teachers Training College University of Yaoundé I Yaoundé Cameroon uy1.uninet.cm; ^3^ The Centre for Research on Medicinal Plants and Traditional Medicine Institute of Medical Research and Medicinal Plants Studies Yaoundé Cameroon impm-cameroun.org; ^4^ Biotechnology Centre University of Yaoundé I Yaoundé Cameroon uy1.uninet.cm; ^5^ Faculty of Science University of Yaoundé I Yaoundé Cameroon uy1.uninet.cm

**Keywords:** hepatic enzymes, lipid profile, *Stereospermum acuminatissimum*, toxicity studies, Wistar rat

## Abstract

**Background:**

Despite its traditional use in Africa for weight management, the safety profile of the aqueous stem bark extract of *Stereospermum acuminatissimum* remains undocumented. This study evaluated its acute and subchronic toxicity in *Wistar* rats.

**Methods:**

Acute toxicity was assessed according to OECD Guideline 425 using a single oral dose of 5000 mg/kg. Subchronic toxicity was evaluated over 56 days at doses of 150, 300 and 600 mg/kg/day. Satellite groups (the control and the 600 mg/kg/day group) were maintained for an additional 14 days to observe the reversibility of any treatment‐related effects. Parameters studied included body/organ weights, haematological, biochemical, lipid and histopathological profiles.

**Results:**

No mortalities or signs of acute toxicity were observed during the 14 days following extract administration. Subchronic administration did not affect survival or relative organ weights, although the 600 mg/kg dose induced minor structural alterations in affected organs. The extract significantly reduced body weight gain. At 150 mg/kg, the extract sex‐specifically improved lipid profiles by lowering triglycerides in males and increasing HDL cholesterol in females. Haematological assessment revealed a dose‐dependent increase in platelet counts in male rats at 600 mg/kg, which returned to baseline levels after the 14‐day recovery period. Elevated AST and ALT fully reversed in females post‐recovery; however, elevated ALT persisted at 600 mg/kg in males. Creatinine remained stable or decreased, and minor variations in urea concentrations in both sexes returned to baseline during recovery.

**Conclusion:**

*S. acuminatissimum* aqueous extract reduces body weight gain and improves lipid profiles at low‐to‐moderate doses (150–300 mg/kg), providing its traditional use. However, the persistence of elevated ALT levels and focal leucocyte infiltration in males at 600 mg/kg indicates high‐dose hepatotoxicity, highlighting a narrow therapeutic window and the need for careful liver function monitoring during prolonged exposure.

## 1. Introduction

*Stereospermum acuminatissimum* (Bignoniaceae) is a medicinal plant of West and Central Africa traditionally used to treat malaria, inflammation, respiratory infections, rheumatism and parasitic diseases [1–7]. Its therapeutic effects have been attributed to a diverse phytochemical composition, including triterpenoids (leutcharic acid), naphthoquinones (pinnatal), anthraquinones (sterequinones) and iridoid glycosides [5, 8, 9].

Several of these metabolites exhibit antibacterial and antitumour activities in vitro. However, evidence of their safety in complex biological systems remains limited. Furthermore, data from systematic in vivo toxicity studies are scarce, and the safety profile of *S. acuminatissimum* has not yet been clearly established. Therefore, the present study was carried out to evaluate the acute and subchronic toxicity of the aqueous stem bark extract of *S. acuminatissimum* in *Wistar* rats and to provide essential safety data to support its potential therapeutic use. While international guidelines, such as OECD Guideline 408, standardise subchronic evaluation over a 90‐day period for full regulatory compliance, preliminary screening frameworks frequently employ intermediate durations to establish initial target‐organ safety. Accordingly, this study was designed as an exploratory evaluation to investigate the intermediate subchronic toxicity profile of the aqueous stem bark extract of *S. acuminatissimum* over a 56‐day (8‐week) exposure period. This screening approach aims to map early cumulative biochemical and histopathological alterations, providing the baseline safety margins required to justify subsequent, full‐scale chronic investigations.

## 2. Materials and Methods

### 2.1. Plant Material

Fresh stem bark of *S. acuminatissimum* K. Schum. was collected in June 2024 from the Bangangté area (West Region, Cameroon). The plant material was authenticated by comparison with Voucher Specimen No. 3011/HNC deposited at the National Herbarium of Cameroon, Yaoundé, Cameroon. The stem bark of *S. acuminatissimum* was shade‐dried at room temperature for four weeks and ground into a fine powder using a mechanical mill.

### 2.2. Plant Extraction

One thousand grams of the powdered stem bark was mixed with 15 L of water to mimic traditional medicinal preparation and boiled for 1 h. After cooling, the mixture was filtered using Whatman No. 3 paper. The filtrate was then evaporated to dryness at 45°C in a Manesty ventilated incubator. A total of 197 g of dry extract was obtained, corresponding to an extraction yield of 19.7%.

### 2.3. Phytochemical Screening

A portion of the aqueous stem bark extract of *S. acuminatissimum* was subjected to phytochemical screening to identify the major classes of secondary metabolites.

All tests were performed in duplicate according to standard procedures: Dragendorff’s/Kraut’s test for alkaloids [10], iodine test for phenolic compounds [11], ferric chloride test for flavonoids [12], Salkowski’s test for terpenoids and sterols [11], Braymer’s test for tannins [13], Borntrager’s test for glycosides [14], Borntrager’s test for anthraquinones [13], NaOH test for coumarins [15], HCl test for anthocyanins [16] and foam test for saponins [14].

### 2.4. Experimental Animals

Male and female *Wistar* strain albino rats were used in this study. Females were nulliparous and non‐pregnant. Male and female rats were housed separately throughout the experimental period. Animals were obtained from the Animal Facility of the Laboratory of Human Metabolism and Non‐Communicable Diseases at the Institute of Medical Research and Medicinal Plants Studies (IMPM). They were housed in well‐ventilated cages lined with wood shavings, which were changed daily, and were provided with a standard diet and tap water *ad libitum*. During the acute and subchronic toxicity studies, rats were housed in groups of three or five animals per cage. Environmental conditions were maintained at 25 ± 3°C under a natural 12‐h light/12‐h dark cycle with adequate ventilation. All experimental procedures and protocols involving animals and their care in this study were conducted in accordance with institutional guidelines and were approved by the National Ethics Committee of Cameroon (Reg. No. FWA‐IRB00001954).

### 2.5. Acute Oral Toxicity Study

Acute toxicity was evaluated according to OECD Guideline 425 using the limit‐test procedure at a dose of 5000 mg/kg body weight [17]. *Wistar* rats of both sexes weighing 183 to 271 g were fasted for 12 h before treatment but had free access to water. The animals were divided into two groups of six rats each (3 males and 3 females). The control group received distilled water (10 mL/kg), whereas the test group received the aqueous extract at 5000 mg/kg. Animals were continuously observed during the first 2 h after administration for signs of toxicity, including changes in appearance, weight, behaviour, and food and water intake, and subsequently monitored daily for two weeks. At the end of the experimental period, the rats were weighed, anaesthetised and autopsied for pathological examination.

### 2.6. Subchronic Oral Toxicity Study

The subchronic toxicity study was conducted over 56 days (8 weeks)—an intermediate duration selected within this exploratory screening framework to evaluate cumulative systemic toxicity and identify primary target organs prior to standard 90‐day regulatory testing. Male and female rats (weighing 160–247 g) were randomly assigned to four primary experimental groups of 10 animals each (5 males and 5 females per group). Control animals received distilled water (10 mL/kg/day), while the treated groups received the aqueous extract of *S. acuminatissimum* at 150, 300 or 600 mg/kg/day via daily oral gavage. Doses were selected based on the previously established anti‐obesity effective dose of 300 mg/kg.

To evaluate the reversibility of potential toxic effects, two additional satellite groups (10 rats each; 5 males and 5 females) were introduced: a satellite control and a high‐dose satellite group (600 mg/kg/day). Following the 56‐day treatment period, satellite animals were maintained for an additional 14 days without treatment. Because male and female data were segregated and analysed independently, the sample size for all subsequent statistical evaluations and data tables is *n* = 5 per sex per group. Body weight was measured throughout the study, and animals were monitored daily for clinical signs of toxicity. Body weight gain was calculated using the following equation:

(1) body weight gain%=final body weight−baseline body weight baseline body weight×100.

#### 2.6.1. Serum Collection and Organ Homogenate Preparation

After a 12‐h fast, the rats were anaesthetised with sodium pentobarbital (50 mg/kg, intraperitoneally) and sacrificed by exsanguination. Blood samples were collected into EDTA tubes for haematological analysis and into plain tubes for biochemical assays. Serum was obtained after centrifugation at 3500 rpm for 10 min. Satellite animals underwent the identical anaesthesia protocol, blood collection and euthanasia protocol on Day 70 following the 14‐day recovery period.

#### 2.6.2. Body Weight and Organ Weight

At the end of the experiment, rats were weighed before sacrifice. The liver, lungs, spleen, heart and gonads were excised and weighed individually using an analytical balance. Following euthanasia, relative organ weights were calculated using the following formula:

relative organ weight (%) = [organ weight (g)/body weight (g)] × 100.

#### 2.6.3. Haematological Analysis

Haematological parameters including haematocrit, haemoglobin concentration, erythrocyte count, white blood cell count, lymphocytes, monocytes and platelet count were assessed using an automated analyser (Beckman Coulter, Thermo Fisher Scientific, United Kingdom).

#### 2.6.4. Biochemical Analysis

Serum alanine aminotransferase (ALT) and aspartate aminotransferase (AST) activities were evaluated using commercial kits from Fortress Diagnostics (Antrim, United Kingdom), whereas creatinine was measured using Biorex Diagnostic Kits (Antrim, United Kingdom). Commercial LABKIT reagents (Barcelona, Spain) were used to determine total cholesterol, HDL cholesterol, triglycerides and urea concentrations. Low‐density lipoprotein (LDL) cholesterol levels were calculated using the formula described by Friedewald et al. [18].

#### 2.6.5. Histopathological Examinations

Liver, kidney and heart were fixed and processed according to standard histological procedures [19]. Paraffin‐embedded tissues were sectioned at a thickness of 4 μm and stained with haematoxylin and eosin (H&E). Histological sections were examined under an Olympus light microscope, and photomicrographs were captured using a camera mounted on the microscope.

### 2.7. Statistical Analysis

Statistical analyses were performed using *R* software Version 4.5.1 and GraphPad Prism 8.0.1 (GraphPad Software, Inc., La Jolla, California, USA). Data normality was assessed using the Shapiro–Wilk test. The results were expressed as means ± standard error of the mean (SEM). One‐way analysis of variance (ANOVA) followed by Tukey’s post hoc test was used when appropriate. Differences were considered statistically significant at *p* < 0.05. Sex‐related differences were taken into consideration during the statistical analyses.

## 3. Results

### 3.1. Phytochemical Screening

Qualitative phytochemical screening of the aqueous extract of *S. acuminatissimum* stem bark revealed the presence of alkaloids, anthraquinones, coumarins, flavonoids, phenolic compounds, saponins, tannins, and terpenoids and sterols (Table 1).

**TABLE 1:** Phytochemical screening of the aqueous extract of *Stereospermum acuminatissimum* stem bark.

Classes of compounds	Results
Alkaloids	+
Anthocyanins	−
Anthraquinones	+
Coumarins	+
Flavonoids	+
Glycosides	−
Phenolic compounds	+
Saponins	+
Tannins	+
Terpenoids, sterols	+

*Note:* (+) presence, (−) absence.

### 3.2. Acute Toxicity

Oral administration of a single dose of 5000 mg/kg of the aqueous extract of *S. acuminatissimum* did not produce any behavioural abnormalities or mortality. Therefore, the median lethal dose (LD_50_) of stem bark aqueous extract of *S. acuminatissimum* was estimated to be greater than 5000 mg/kg. Body weight increased slightly in both male and female treated groups, but the changes were not significantly different from those in the respective control groups. No alterations in coat appearance or colour, locomotor activity, response to noise, aggressive behaviour, or food and water intake were observed during the first 4 h and throughout the 14‐day period following extract administration.

### 3.3. Subchronic Toxicity

#### 3.3.1. Body Weight Change (%)

As shown in Table 2, body weight gain in females was comparable to that of controls between Day 14 and Day 28. However, from Day 35 onward, animals treated with 150, 300 and 600 mg/kg exhibited significantly lower body weight gain than the control group. A significant reduction in body weight gain in females was observed on Day 35 in the 150 mg/kg group (10.57 ± 1.5% compared to 19.61 ± 2.31% in the control group). By Day 49, females receiving 150 mg/kg exhibited a weight gain of 9.90 ± 0.51% vs. 27.38 ± 2.57% in the control group. Similarly, male rats treated with 150 mg/kg showed significantly lower body weight gain than controls on Day 49 (13.49 ± 1.13% vs. 42.88 ± 2.37%). On Day 56, both males and females receiving the extract exhibited significantly lower body weight gain than their respective controls, irrespective of dose. The extract‐treated satellite groups also displayed reduced body weight gain. By Day 56, female satellite rats exhibited a lower increase in body weight than the normal satellite group (12.77 ± 0.52% vs. 26.89 ± 1.97%). Similarly, in male satellite rats, reduced body weight gain persisted up to Day 70, reaching 43.77 ± 2.53% compared to 70.48 ± 7.11% in the normal satellite group.

**TABLE 2:** Effects of the aqueous extract of *Stereospermum acuminatissimum* stem bark on weekly body weight changes in male and female rats in subchronic toxicity.

Sex/Treatment	D0	D7	D14	D21	D28	D35	D42	D49	D56	D63	D70
*Female*											
NC	0	2.42 ± 0.32	9.56 ± 0.49^b^	12.58 ± 0.85^b^	13.31 ± 2.57	19.61 ± 2.31^a^	22.36 ± 2.33^a^	25.10 ± 2.52^b^	27.38 ± 2.57^b^		
E150	0	1.15 ± 1.25	7.01 ± 1.48^ab^	10.73 ± 1.80^ab^	11.65 ± 1.50	10.57 ± 1.5^b^	8.97 ± 1.43^c^	8.49 ± 0.67^a^	9.90 ± 0.51^a^		
E300	0	1.14 ± 0.41	8.57 ± 1.38^b^	8.90 ± 0.30^ab^	11.81 ± 1.25	16.43 ± 1.40^ab^	14.48 ± 1.09^bc^	12.91 ± 1.47^a^	11.19 ± 1.34^a^		
E600	0	2.74 ± 0.53	9.46 ± 0.45^b^	12.32 ± 0.88^b^	12.65 ± 0.97	14.54 ± 0.85^ab^	15.36 ± 0.92^b^	13.87 ± 1.30^a^	11.32 ± 1.22^a^		
NS	0	1.63 ± 0.78	4.06 ± 0.55^a^	7.43 ± 0.67^a^	10.15 ± 1.49	13.61 ± 1.34^ab^	17.36 ± 1.42^ab^	20.07 ± 1.58^b^	26.89 ± 1.97^b^	35.07 ± 2.03	43.81 ± 2.38
SE600	0	1.44 ± 0.36	6.17 ± 0.60^ab^	9.68 ± 0.49^ab^	11.48 ± 0.57	13.67 ± 0.55^ab^	11.70 ± 0.56^bc^	10.05 ± 0.53^a^	12.77 ± 0.52^a^	27.80 ± 2.45	36.59 ± 3.29

*Male*											
NC	0	5.32 ± 0.78	12.92 ± 1.37	18.37 ± 0.91	20.85 ± 0.51	30.98 ± 1.31^ab^	37.43 ± 1.33^ab^	42.88 ± 2.37^ac^	48.88 ± 2.25^a^		
E150	0	2.90 ± 0.58	11.49 ± 1.18	20.31 ± 0.57	22.20 ± 0.71	20.42 ± 0.77^b^	17.56 ± 0.60^b^	13.49 ± 1.13^b^	11.76 ± 0.93^c^		
E300	0	6.9 ± 4.85	14.88 ± 4.86	20.11 ± 4.66	26.37 ± 5.22	23.85 ± 4.76^ab^	21.92 ± 4.96^b^	20.12 ± 5.20^bc^	19.03 ± 5.16^bc^		
E600	0	3.01 ± 0.67	9.96 ± 1.41	15.46 ± 1.87	17.11 ± 1.49	17.62 ± 1.87^b^	17.82 ± 2.42^b^	14.08 ± 1.94^bc^	11.54 ± 2.18^c^		
NS	0	6.05 ± 0.46	18.30 ± 2.08	24.54 ± 3.11	28.53 ± 4.76	39.46 ± 6.09^a^	52.24 ± 7.59^a^	53.27 ± 5.89^a^	63.77 ± 7.58^a^	70.48 ± 7.11^b^	70.48 ± 7.11^b^
SE600	0	4.64 ± 0.43	11.29 ± 0.53	14.60 ± 0.61	14.84 ± 1.24	23.61 ± 0.83^ab^	24.45 ± 1.45^ab^	24.34 ± 3.70^ab^	33.05 ± 2.30^ab^	43.77 ± 2.53^a^	43.77 ± 2.53^a^

*Note:* Data are presented as mean ± SEM (*n* = 5). Means within a column with different superscript letters (a, b, c) differ significantly by Tukey’s HSD test (*p* < 0.05), while means sharing a common letter or without superscripts are not significantly different (*p* > 0.05). NS, normal satellite control; E150, E300, E600, *S. acuminatissimum* extract groups (150, 300, 600 mg/kg); SE600, extract satellite group (600 mg/kg). D63 and D70 apply exclusively to the satellite groups (NS and SE600), as the main study groups were sacrificed on Day 56.

Abbreviation: NC, normal control.

#### 3.3.2. Relative Organ Weight

Oral administration of the aqueous stem bark extract of *S. acuminatissimum* did not significantly affect the relative weights of the liver, lungs, spleen, heart, or gonads in either sex. The data revealed that the carcass yields of the normal control (NC) and extract‐treated groups (E150, E300, and E600 receiving 150, 300, and 600 mg/kg/day, respectively) were similar, with carcass yields of approximately 76%–77% (g/100 g b.w.), showing no statistically significant differences. Conversely, significantly higher values (∼79%, denoted by superscript “b”) were observed in the normal satellite control (NS) andrecovery satellite group (SE600) groups where treatment was interrupted two weeks after the experiment (Table 3).

**TABLE 3:** Effects of the aqueous extract of *Stereospermum acuminatissimum* stem bark on relative organ weight (g/100 g).

Sex/Organ	NC	E150	E300	E600	NS	SE600
*Female*						
Liver	3.04 ± 0.14	3.53 ± 0.19	3.28 ± 0.13	3.46 ± 0.12	3.09 ± 0.03	3.08 ± 0.12
Kidneys	0.55 ± 0.03	0.54 ± 0.03	0.53 ± 0.00	0.53 ± 0.01	0.49 ± 0.08	0.50 ± 0.00
Spleen	0.22 ± 0.00	0.23 ± 0.03	0.23 ± 0.01	0.21 ± 0.01	0.23 ± 0.00	0.21 ± 0.00
Heart	0.34 ± 0.01	0.32 ± 0.01	0.35 ± 0.01	0.34 ± 0.01	0.34 ± 0.01	0.33 ± 0.01
Lungs	0.78 ± 0.10	0.69 ± 0.03	0.68 ± 0.06	0.70 ± 0.04	0.60 ± 0.08	0.75 ± 0.08
Ovaries	0.06 ± 0.00	0.07 ± 0.00	0.07 ± 0.01	0.07 ± 0.01	0.07 ± 0.00	0.06 ± 0.00
Carcass	76.14 ± 0.90	75.46 ± 1.12	76.67 ± 0.81	79.99 ± 0.46	78.99 ± 0.46	79.85 ± 0.93

*Male*						
Liver	3.58 ± 0.04	3.59 ± 0.10	3.50 ± 0.15	3.58 ± 0.09	3.89 ± 0.10	3.47 ± 0.19
Kidneys	0.49 ± 0.02	0.52 ± 0.01	0.53 ± 0.01	0.54 ± 0.01	0.54 ± 0.00	0.53 ± 0.03
Spleen	0.24 ± 0.00	0.20 ± 0.01	0.20 ± 0.00	0.18 ± 0.01	0.19 ± 0.01	0.22 ± 0.03
Heart	0.28 ± 0.01	0.31 ± 0.01	0.24 ± 0.04	0.32 ± 0.01	0.34 ± 0.01	0.31 ± 0.00
Lungs	0.60 ± 0.05	0.50 ± 0.02	0.55 ± 0.01	0.58 ± 0.25	0.65 ± 0.04	0.57 ± 0.05
Testicles	0.97 ± 0.02	1.09 ± 0.03	1.03 ± 0.11	1.17 ± 0.03	1.18 ± 0.04	1.16 ± 0.04
Carcass	76.19 ± 0.64^a^	76.32 ± 2.12^a^	77.48 ± 0.57^a^	77.48 ± 0.61^a^	79.59 ± 0.52^b^	79.34 ± 0.77^b^

*Note:* Values are expressed as mean ± SEM (*n* = 5). Within the same row, means with different superscript letters (a, b) are statistically different based on Tukey’s HSD post hoc test (*p* < 0.05), while means sharing a common letter or without superscripts are not significantly different (*p* > 0.05). NS: normal satellite control; E150, E300 and E600: groups treated with *Stereospermum acuminatissimum* aqueous extract at 150, 300 and 600 mg/kg/day, respectively; SE600: satellite group treated with 600 mg/kg/day.

Abbreviation: NC, normal control.

#### 3.3.3. Haematological Parameters

The effects of the aqueous stem bark extract of *S. acuminatissimum* on haematological markers are illustrated in Table 4. In female rats, white blood cell and lymphocyte counts followed similar patterns. The E300 group exhibited higher peaks (6.51 ± 0.44 for WBC and 5.36 ± 0.48, respectively) than the recovery satellite group SE600 (3.96 ± 0.04 and 2.68 ± 0.16, respectively). However, no significant differences were observed between the normal control (NC) group and the treated groups (E150, E300, E600). Platelet counts remained unaffected irrespective of the dose. White blood cell, lymphocyte, monocyte, red blood cell and haemoglobin counts in male rats were not significantly altered by treatment irrespective of the group. In contrast, platelet counts exhibited a dose‐dependent increase, reaching 904.6 ± 46.62 × 10^3^/μL in the E600 group. This value was significantly higher than that observed in the recovery satellite group SE600 (484 ± 9.85).

**TABLE 4:** Effects of the aqueous extract of *Stereospermum acuminatissimum* stem bark on haematological parameters of male and female rats.

Sex/Parameters	NC	E150	E300	E600	NS	SE600
*Female*						
WBC (10^3^/μL)	5.02 ± 0.42^bc^	4.26 ± 0.44^bc^	6.51 ± 0.44^c^	5.71 ± 0.17^ac^	3.78 ± 0.73^ab^	3.96 ± 0.04^b^
LYM (10^3^/μL)	3.80 ± 0.28^bc^	3.12 ± 0.36^ab^	5.36 ± 0.48^c^	4.61 ± 0.24^ac^	2.94 ± 0.68^ab^	2.68 ± 0.16^b^
MON (10^3^/μL)	0.99 ± 0.15	0.99 ± 0.08	1.01 ± 0.31	0.98 ± 0.16	0.89 ± 0.32	0.66 ± 0.13
RBC (10^6^/μL)	8.46 ± 0.17	8.74 ± 0.19	8.52 ± 0.08	8.69 ± 0.16	8.61 ± 0.23	8.34 ± 0.27
HGB (g/L)	14.55 ± 0.12	14.74 ± 0.15	14.86 ± 0.16	14.96 ± 0.18	14.9 ± 0.33	14.45 ± 0.32
PLT (10^3^/μL)	774.75 ± 109.71	886.6 ± 58.20	934.8 ± 42.47	917.8 ± 28.34	895 ± 8.78	779.50 ± 63.51

*Male*						
WBC (10^3^/μL)	3.20 ± 0.19	3.94 ± 0.82	3.39 ± 0.15	8.36 ± 3.46	6.56 ± 1.78	2.2 ± 0.13
LYM (10^3^/μL)	2.72 ± 0.16	2.68 ± 0.57	2.80 ± 0.19	4.74 ± 1.11	5.44 ± 1.44	2.48 ± 0.51
MON (10^3^/μL)	0.41 ± 0.16	0.15 ± 0.01	0.34 ± 0.06	2.98 ± 2.25	0.93 ± 0.24	1.36 ± 0.43
RBC (10^6^/μL)	8.59 ± 0.39	9.43 ± 0.05	9.37 ± 0.06	9.03 ± 0.36	8.89 ± 0.12	8.99 ± 0.13
HGB (g/L)	15.05 ± 0.61	16.1 ± 0.24	16.08 ± 0.04	15.4 ± 0.52	15.78 ± 0.26	15.80 ± 0.25
PLT (10^3^/μL)	546.75 ± 134.31^ab^	718.20 ± 60.60^ab^	800.6 ± 34.47^ab^	904.6 ± 46.62^b^	779.8 ± 67.49^ab^	484 ± 9.85^a^

*Note:* Values are expressed as mean ± SEM (*n* = 5). Different superscript letters (a, b, c) within the same row indicate statistically significant differences (*p* < 0.05) based on Tukey’s HSD post hoc test; means sharing a common letter or without superscripts do not differ significantly (*p* > 0.05). LYM: lymphocytes; MON: monocytes; HGB: haemoglobin; PLT: platelets. NS: normal satellite control; E150, E300, E600: groups treated with *Stereospermum acuminatissimum* aqueous extract at 150, 300 and 600 mg/kg/day, respectively; SE600: satellite recovery group (600 mg/kg/day).

Abbreviations: NC, normal control; RBC, red blood cell; WBC, white blood cell.

#### 3.3.4. Biochemical Analysis

Subchronic administration of 600 mg/kg of the extract significantly increased serum AST activity in female rats from 4.80 ± 0.65 IU/L in the control group to 12.22 ± 1.23 IU/L (Figure 1A; *p* < 0.05). However, following the two‐week recovery period, AST levels in the SE600 satellite group significantly declined to 3.05 ± 0.34 IU/L, which was lower than that observed both during treatment and in the normal satellite group (7.71 ± 0.68 IU/L; *p* < 0.001; Figure 1B). No significant effects on AST activity were recorded in male rats irrespective of the dosage (Figure 1C,D).

**FIGURE 1:**
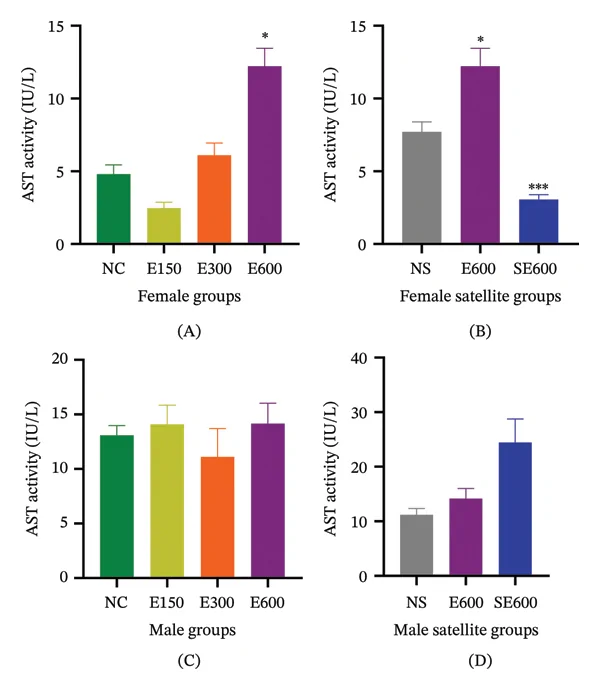
Effects of the *Stereospermum acuminatissimum* stem bark aqueous extract on aspartate aminotransferase activity in female (A, B) and male (C, D) rats. Note: Each bar represents the mean value ± SEM. ^∗^ *p* < 0.05; ^∗∗∗^ *p* < 0.001: significant difference compared to the normal control (NC) and normal satellite (NS) groups. E150, E300 and E600: groups treated with aqueous extract of *Stereospermum acuminatissimum* at doses of 150, 300 and 600 mg/kg, respectively. SE600: satellite group treated with 600 mg/kg.

In females, ALT levels following subchronic treatment at 600 mg/kg were significantly elevated, increasing from 7.33 ± 1.16 IU/L to 14.55 ± 0.32 IU/L (Figure 2A; *p* < 0.05). No significant differences were noted between the female satellite groups after the recovery period (Figure 2B). In males, ALT activity increased significantly (*p* < 0.01) at 300 and 600 mg/kg, reaching 15.71 ± 0.55 IU/L (*p* < 0.001) and 12.80 ± 1.15 IU/L, respectively, compared to 6.69 ± 0.69 IU/L in controls (Figure 2C). Following the recovery period, ALT activity remained elevated in the SE600 satellite group (24.59 ± 3.90 IU/L) compared with the normal satellite group (6.28 ± 1.05 IU/L; *p* < 0.001; Figure 2D).

**FIGURE 2:**
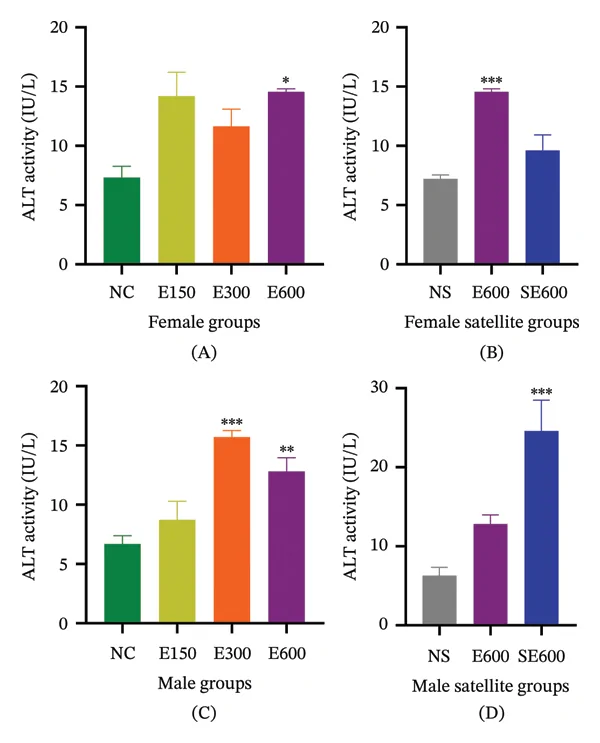
Effects of *Stereospermum acuminatissimum* bark aqueous extract on alanine aminotransferase activity in female (A, B) and male (C, D) rats. Note: Each bar represents the mean value ± SEM. ^∗^ *p* < 0.05; ^∗∗^ *p* < 0.01; ^∗∗∗^ *p* < 0.001: significant differences compared to the normal control (NC) and normal satellite (NS) groups. E150, E300 and E600: groups treated with *Stereospermum acuminatissimum* aqueous extract at doses of 150, 300 and 600 mg/kg, respectively. SE600: satellite group treated with 600 mg/kg.

In females, serum creatinine levels decreased in a dose‐dependent manner, reaching 0.20 ± 0.02 mg/dL at the 600 mg/kg dose (*p* < 0.01; Figure 3A). After the two‐week recovery period, creatinine levels increased to 0.34 ± 0.05 mg/dL in the SE600 group but remained significantly lower than those in the normal satellite controls (*p* < 0.05; Figure 3B). In males, no significant changes occurred during the treatment phase (Figure 3C). However, after the recovery period, the male satellite group (SE600) exhibited significantly lower creatinine levels (0.23 ± 0.02 mg/dL) than the normal satellite group (0.48 ± 0.03 mg/dL; *p* < 0.001; Figure 3D).

**FIGURE 3:**
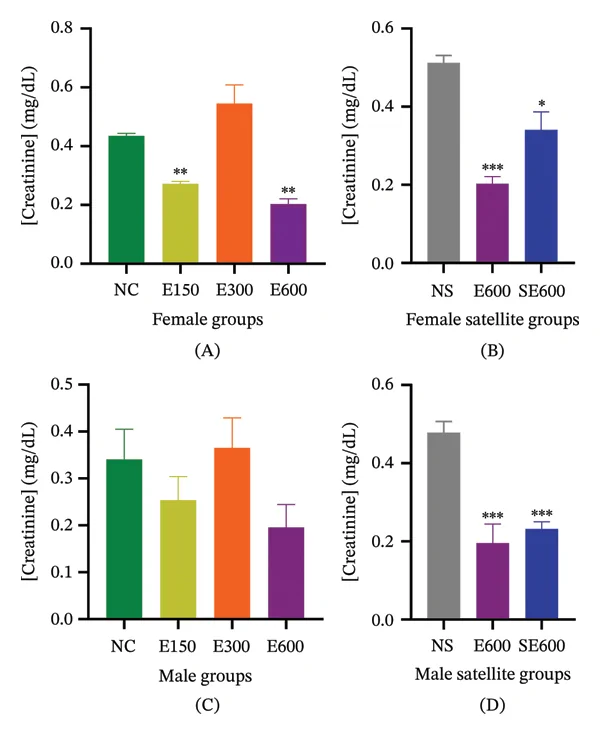
Effects of *Stereospermum acuminatissimum* bark aqueous extract on serum creatinine levels in female (A, B) and male (C, D) rats. Note: Each bar represents the mean value ± SEM. ^∗^ *p* < 0.05; ^∗∗^ *p* < 0.01; ^∗∗∗^ *p* < 0.001: significant difference compared to the normal control (NC) and normal satellite (NS) groups. E150, E300 and E600: groups treated with *Stereospermum acuminatissimum* aqueous extract at doses of 150, 300 and 600 mg/kg, respectively. SE600: satellite group treated with 600 mg/kg.

In females, serum urea concentrations decreased significantly in a dose‐dependent manner, with the most pronounced reduction occurring at 300 mg/kg (19.41 ± 2.61 mg/dL) compared with the controls (51.94 ± 5.55 mg/dL) (*p* < 0.01; Figure 4A). These values returned to normal following the recovery period in the satellite groups (Figure 4B).

**FIGURE 4:**
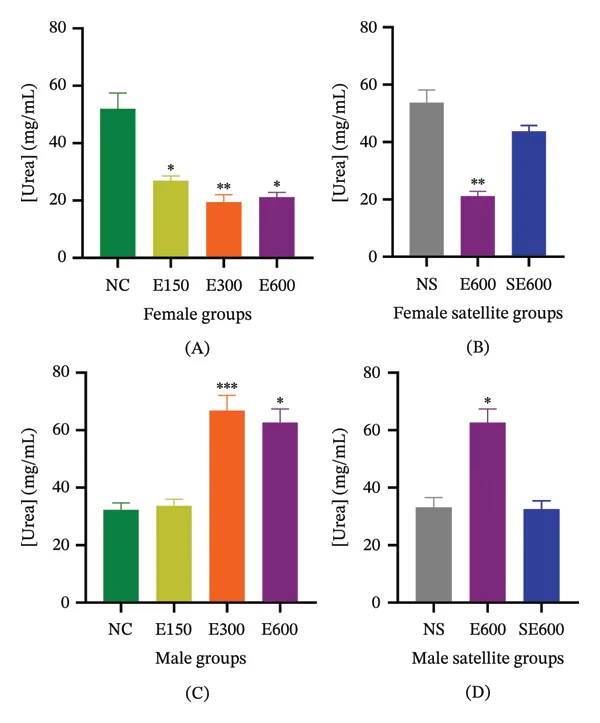
Effects of *Stereospermum acuminatissimum* bark aqueous extract on serum urea levels in female (A, B) and male (C, D) rats. Note: Each bar represents the mean value ± SEM. ^∗^ *p* < 0.05; ^∗∗^ *p* < 0.01; ^∗∗∗^ *p* < 0.001: significant difference compared to the normal (NC) and normal satellite (NS) control groups. E150, E300 and E600: groups treated with *Stereospermum acuminatissimum* aqueous extract at doses of 150, 300 and 600 mg/kg, respectively. SE600: satellite group treated with 600 mg/kg.

In contrast, in males, serum urea concentrations increased significantly at 300 mg/kg and 600 mg/kg, reaching 66.82 ± 5.29 mg/dL and 62.63 ± 4.73 mg/dL, respectively, compared with the NC group (Figure 4C; *p* < 0.05). No significant differences were observed in the satellite groups after the recovery period (Figure 4D).

As shown in Table 5, female rats receiving 150 mg/kg exhibited significantly higher HDL cholesterol levels (127.10 ± 7.49 mg/dL) than the NC group (77.52 ± 9.11 mg/dL) and all other treatment groups (E300, E600, NS and SE600). In male rats, triglyceride concentrations were significantly lower at 150 mg/kg (139.81 ± 19.04 mg/dL) than the control (269.44 ± 19.91 mg/dL), reaching values comparable to those observed in the SE600 satellite group (124.07 ± 1.54 mg/dL). In contrast, no significant triglyceride concentrations were observed among the E300, E600 and NS groups (Table 5).

**TABLE 5:** Effects of aqueous extract of *Stereospermum acuminatissimum* bark on lipid profiles in female and male rats.

Sex/Parameters (mg/dL)	NC	E150	E300	E600	NS	SE600
*Female*						
TOT‐CHOL	127.08 ± 7.04	155.22 ± 8.00	153.58 ± 17.85	180.59 ± 17.90	134.17 ± 13.68	145.99 ± 22.24
TG	236.72 ± 35.66	243.74 ± 13.26	236.11 ± 59.44	266.66 ± 34.50	269.90 ± 39.67	277.77 ± 11.65
HDL‐CHOL	77.52 ± 9.11^b^	127.10 ± 7.49^a^	93.80 ± 16.51^b^	111.50 ± 10.64^b^	84.51 ± 15.93^b^	101.23 ± 10.40^b^
LDL‐CHOL	19.68 ± 2.66	12.70 ± 1.03	12.55 ± 19.95	14.16 ± 0.05	6.50 ± 3.29	13.15 ± 7.14

*Male*						
TOT‐CHOL	180.06 ± 10.71	143.67 ± 5.39	175.94 ± 24.44	153.16 ± 12.84	157.59 ± 16.76	144.30 ± 8.24
TG	269.44 ± 19.91^b^	139.81 ± 19.04^a^	296.29 ± 19.06^b^	278.70 ± 38.62^b^	287.03 ± 41.57^b^	124.07 ± 1.54^a^
HDL‐CHOL	99.99 ± 11.20	99.11 ± 8.11	112.38 ± 7.17	87.61 ± 3.07	107.61 ± 18.60	94.91 ± 11.96
LDL‐CHOL	31.05 ± 7.44	23.32 ± 8.91	21.30 ± 10.95	31.60 ± 12.99	25.84 ± 1.97	17.11 ± 5.75

*Note:* Each value represents the mean ± SEM (*n* = 5). Means within the same row bearing different superscript letters (a, b) are significantly different (*p* < 0.05) based on Tukey’s HSD post hoc test, while means sharing a common letter or without superscripts are not significantly different (*p* > 0.05). NS: normal satellite control; E150, E300 and E600: groups treated with *Stereospermum acuminatissimum* aqueous extract at 150, 300 and 600 mg/kg/day, respectively; SE600: satellite group treated with 600 mg/kg/day. TOT‐CHOL: total cholesterol; TG: triglycerides; HDL‐CHOL: high‐density lipoprotein cholesterol; LDL‐CHOL: low‐density lipoprotein cholesterol.

Abbreviation: NC, normal control.

#### 3.3.5. Histopathological Evaluation

Figures 5 and 6 present the effects of the extract on the architecture of the liver, kidneys and heart of female and male rats, respectively. Leucocyte infiltration (IL) was observed in the liver of female rats receiving 300 and 600 mg/kg (Figure 5). In male rats, liver sections from the control and lower dose groups showed a normal hepatic architecture. However, mild, focal IL was noted at the highest dose (600 mg/kg), while the underlying lobular structure remained well‐defined (Figure 6). Kidney sections from both sexes revealed normal histological features, characterised by glomeruli and distinct urinary spaces. Likewise, cardiac tissues showed normal architecture with distinct muscle fibres and nuclei in all groups.

**FIGURE 5:**
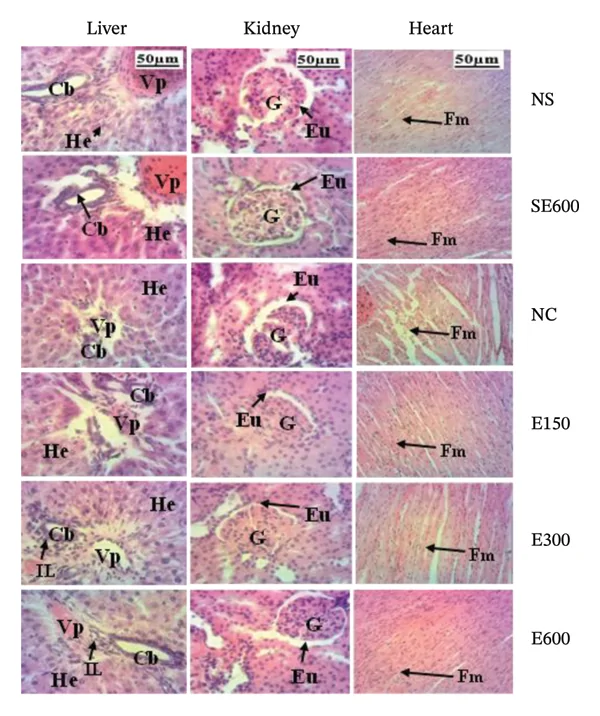
Effects of the extract on the architecture of the liver, kidney and heart of female rats. Note: liver: Vp = portal vein, He = hepatocyte, Cb = bile canaliculus, IL = leucocyte infiltration; kidney: G = glomerulus, Eu = urinary space; Heart: N = nucleus, Fm = muscle fibre. X: 100X (Heart) and 250X (kidney and liver). Normal control (NC) and normal satellite (NS) control groups. E150, E300 and E600: groups treated with the aqueous extract of *S. acuminatissimum* at doses of 150, 300 and 600 mg/kg, respectively. SE600: satellite group treated with 600 mg/kg.

**FIGURE 6:**
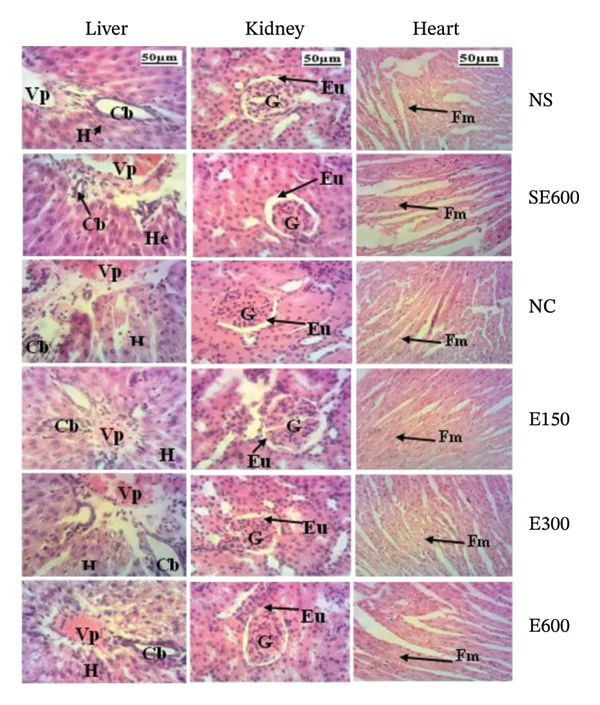
Effects of the extract on the architecture of the liver, kidney and heart of male rats. Note: liver: Vp = portal vein, He = hepatocyte, Cb = bile canaliculus, IL = leucocyte infiltration; kidney: G = glomerulus, Eu = urinary space; heart: N = nucleus, Fm = muscle fibre. X: 100X (heart) and 250X (kidney and liver). Normal control (NC) and normal satellite (NS) control groups. E150, E300 and E600: Groups treated with aqueous extract of *Stereospermum acuminatissimum* at doses of 150, 300 and 600 mg/kg, respectively. SE600: satellite group treated with 600 mg/kg.

## 4. Discussion

*S. acuminatissimum* is traditionally used in Cameroon for the management of several diseases [2]. The present study evaluated the acute toxicity of the aqueous stem bark extract of *S. acuminatissimum* and investigated the effects of its subchronic administration on body weight and haematological parameters, lipid profiles, hepatorenal biomarkers and the histopathology of the heart, liver and kidneys.

Acute oral administration of the extract at a dose of 5000 mg/kg produced no signs of toxicity or mortality in *Wistar* rats. Since LD_50_ was greater than 5 g/kg, the extract may be considered to possess low acute toxicity according to OECD criteria [17].

The selected doses (150, 300 and 600 mg/kg) for subchronic exposure were based on an effective anti‐obesity dose of 300 mg/kg identified in a preliminary investigation. Total body and organ weights are routinely assessed in toxicological studies because changes in body weight are among the earliest and most sensitive indicators of adverse treatment effects induced by xenobiotics [20]. The results of the subchronic study showed that the aqueous extract significantly reduced weight gain in both male and female rats from Day 35 onwards. This effect was dose‐dependent, sustained throughout the treatment period and persisted after treatment withdrawal, particularly at the highest dose. These findings suggest that the extract may influence energy metabolism and/or appetite regulation.

It is possible that the weight‐lowering effects observed are mediated by specific triterpenoids or phenolic derivatives inherently present in *Stereospermum* species. Previous studies have highlighted the anti‐obesity properties of isolated compounds such as ursolic acid and ellagic acid [21–23]; however, further quantitative HPLC‐MS analysis is required to confirm whether these specific active principles are present in our particular aqueous stem bark extract.

The plant extract demonstrated a good safety profile regarding general metabolism during the active treatment phase. Specifically, the carcass weight at the highest dose of 600 mg/kg (E600: 77.48 ± 0.61/100 g bw) was statistically comparable to the NC group (NC: 76.19 ± 0.64/100 g bw, *p* > 0.05), indicating that subchronic consumption of the extract does not induce muscular atrophy, pathological wasting or abnormal adiposity. Furthermore, the observed increase in carcass weight in the satellite groups, NS (79.59 ± 0.52/100 g bw) and SE600 (79.34 ± 0.77/100 g bw) is most likely due to a time‐dependent physiological growth curve rather than a direct effect of the treatment. This increase in body weight is likely attributable to the longer observation period associated with the reversibility protocol and confirms that the 600 mg/kg extract treatment did not permanently alter or stunt the natural growth trajectory of the animals, as the SE600 group exhibited values comparable to those observed in the NS group.

The observed increase in male rat carcass weight following treatment cessation may reflect compensatory physiological adaptations [24]. Alternatively, this gain could reflect the disappearance of a transient inhibitory effect [25] or a metabolic recovery period that improves feed conversion into muscle mass [26].

Haematological analysis revealed a clear, dose‐dependent upward trend in platelet counts among male rats, peaking at the 600 mg/kg dose (904.6 ± 46.62 × 10^3^/μL) compared with the NC group(546.75 ± 134.31 × 10^3^/μL). Although this elevation did not achieve statistical significance relative to the control group due to within‐group variance, it was significantly different from the satellite recovery group (484 ± 9.85 × 10^3^/μL), demonstrating that this transient physiological shift fully reversed after treatment cessation.

In this study, subchronic oral administration of the *S. acuminatissimum* extract at the highest dose (600 mg/kg/day) revealed signs of hepatotoxicity, particularly in the male group. This is evidenced by a highly significant elevation of serum ALT levels (*p* < 0.001), which persisted exclusively in this group—and even progressed (24.59 ± 3.90 IU/L)—following the 14‐day treatment‐free recovery period (Figure 2D).

Critically, these biochemical alterations correlate directly with our histopathological findings, which demonstrated focal IL in the liver tissue of both sexes at elevated doses (Figures 5 and 6). Rather than representing transient physiological stress, the concomitant rise in leakage enzymes (ALT and AST) and microscopic inflammatory cell infiltration suggest low‐grade, localised hepatocellular injury or an ongoing cell‐mediated immune response induced by the extract at high concentrations.

The persistence of this damage exclusively in males suggests a sex‐dependent susceptibility, which may be related to sex‐dependent differences in xenobiotic metabolism.

The findings align in part with those of Jada et al. [27], who reported changes in serum transaminases following the administration of the *S. kunthianum* extract. In contrast, *S. acuminatissimum* contains iridoid glycosides, which are known to provide hepatoprotective effects through antioxidant, anti‐inflammatory and antiapoptotic mechanisms. These compounds have been reported to protect against non‐alcoholic fatty liver disease (NAFLD) and fibrosis through the modulation of the Nrf2 and NF‐κB signalling pathways and improvement of mitochondrial function and lipid metabolism [28, 29].

The serum creatinine levels indicated sex‐dependent responses to subchronic administration of the aqueous extract of *S. acuminatissimum*. In female rats, creatinine levels significantly decreased at doses of 150 and 600 mg/kg (*p* < 0.01) and tended to return toward normal after treatment cessation, suggesting recovery following discontinuation. In contrast, the reduction observed in the male satellite group persisted after the recovery period, suggesting sustained metabolic changes or persistent modulation of renal function.

Serum urea concentrations also showed sex‐dependent variations. In female rats, urea levels decreased significantly across all treatment groups (150, 300 and 600 mg/kg), with a more pronounced reduction at 300 mg/kg (*p* < 0.05). This reduction may partly suggest an early impairment of liver function and could be associated with the inflammatory changes observed in liver sections. Conversely, in male rats, the administration of the extract induced a significant increase in urea levels at doses of 300 and 600 mg/kg, signalling potential alterations in urinary excretion or urea accumulation. Importantly, the increase in serum urea in males was not accompanied by elevated creatinine concentrations or histological abnormalities in the kidneys. Consequently, these changes are more likely to reflect functional or prerenal factors rather than structural renal damage. Similar alterations in renal function parameters have been reported for *S. kunthianum* [30].

Lipid profile analysis revealed both sex‐ and dose‐dependent effects. Female rats treated with 150 mg/kg exhibited an increase in HDL cholesterol, suggesting a potential cardioprotective effect at lower concentrations. In male rats, triglyceride levels decreased significantly at 150 mg/kg, indicating a lipid‐lowering effect at low doses. These findings support the anti‐obesity activity suggested by preliminary studies and indicate that the aqueous extract of *S. acuminatissimum* may modulate lipid metabolism.

The study adopted a 56‐day exposure, shorter than the OECD 90‐day protocol, to assess early‐to‐midterm systemic toxicity and minimise age‐related confounding factors in rodents. Most hepatocellular, renal and haematological injuries occur within 4–8 weeks, and this period effectively reveals cumulative toxicity, organ vulnerability and metabolic adaptations. The findings provide a preliminary safety profile of *S. acuminatissimum* and help identify potential target organs after repeated administration. However, the reduced duration precludes statements about delayed effects, and thus, definitive safety margins for lifetime use will require full 90‐day and chronic studies.

## 5. Conclusions

The aqueous stem bark extract of *S. acuminatissimum* exhibited a favourable acute safety profile, with an estimated LD_50_ greater than 5000 mg/kg. In the subchronic study, its administration revealed a distinct dose‐dependent toxicological profile. While lower doses were generally well tolerated under the conditions of the present study, and show promising metabolic and lipid‐lowering benefits, subchronic exposure to a high dose (600 mg/kg/day) induces persistent hepatic inflammation and enzyme leakage in male rats. Therefore, while the extract holds therapeutic potential, its safety appears to be confined to lower dose ranges, and a definitive no observed adverse effect level (NOAEL) must be strictly established below 600 mg/kg/day before clinical translation.

## Author Contributions

Fabrice Mba Medou: writing–original draft, methodology, investigation, funding acquisition, formal analysis, data curation and conceptualisation. Lucienne Armelle Brooks: methodology, investigation and formal analysis. Nyemb Nyunaï: writing–review and editing, validation, supervision, conceptualisation and project administration. Leonel Ntepe Mbah: methodology and formal analysis. Esther Ngo Lemba Tom: writing–original draft, review and editing, supervision, investigation and conceptualisation. Theodora Kowa Kopa: investigation. Rosette Megnekou: review and editing, validation, supervision, conceptualisation and project administration.

## Funding

This research did not receive any specific grant from funding agencies in the public, commercial or not‐for‐profit sectors.

## Conflicts of Interest

The authors declare no conflicts of interest.

## Data Availability

The data used to support the findings of this study are available from the corresponding author upon request.
